# Combined kyphoplasty and intraoperative radiotherapy (Kypho-IORT) versus external beam radiotherapy (EBRT) for painful vertebral metastases - a randomized phase III study

**DOI:** 10.1186/s12885-019-5666-5

**Published:** 2019-05-09

**Authors:** Frederic Bludau, Grit Welzel, Tina Reis, Yasser Abo-Madyan, Elena Sperk, Frank Schneider, Sven Clausen, Arne M. Ruder, Udo Obertacke, Maged M. Ghaly, Frederik Wenz, Frank A. Giordano

**Affiliations:** 1 Department of Orthopaedic and Trauma Surgery, University Medical Center Mannheim, University of Heidelberg, Mannheim, Germany; 2 Department of Radiation Oncology, University Medical Center Mannheim, University of Heidelberg, Theodor-Kutzer-Ufer 1-3, 68167 Mannheim, Germany; 3 Department of Radiation Medicine, Northwell Health Physician Partners, Hofstra Northwell School of Medicine, Lake Success, NY USA

**Keywords:** Kypho-IORT, Intraoperative radiotherapy, Kyphoplasty, Cement augmentation, Vertebral metastases, External beam radiotherapy

## Abstract

**Background:**

The spine is the most frequent location of bone metastases. Local treatment aims at palliation of pain and, given the increased likelihood of long-term cancer survival, at local control. Kyphoplasty and intraoperative radiotherapy (Kypho-IORT) provided instantaneous pain relief in 70% of patients at the first day after the intervention and resulted in local control rates of > 93% at 1 year in a recently conducted phase I/II trial. To assess its clinical value, we designed a phase III trial which tests Kypho-IORT against the most widespread standard-of-care, external beam radiotherapy (EBRT), in patients with painful vertebral metastases.

**Methods:**

This phase III study includes patients ≥50 years of age with up to 4 vertebral metastases and a pain score of at least 3/10 points on the visual/numeric analogy scale (VAS). Patients randomized into the experimental arm (A) will undergo Kypho-IORT (Kyphoplasty plus IORT with 8 Gy prescribed to 13 mm depth). Patients randomized into the control arm (B) will receive EBRT with either 30 Gy in 10 fractions or 8 Gy as a single dose. The primary end point is pain reduction defined as at least − 3 points on the VAS compared to baseline at day 1. Assuming that 40% of patients in the Kypho-IORT arm and 5% of patients in the control arm will achieve this reduction and 20% will drop out, a total of 54 patients will have to be included to reach a power of 0.817 with a two-sided alpha of 0.05. Secondary endpoints are evaluation of the percentage of patients with a pain reduction of at least 3 points at 2 and 6 weeks, local tumor control, frequency of re-intervention, secondary fractures/sintering, complication rates, skin toxicity/wound healing, progression-free survival (PFS), overall survival (OS) and quality of life.

**Discussion:**

This trial will generate level 1 evidence on the clinical value of a one-stop procedure which may provide instantaneous pain relief, long-term control and shortened intervals to further adjuvant (systemic) therapies in patients with spinal metastases.

**Trial registration:**

Registered with ClinicalTrials.gov, number: NCT02773966 (Registration date: 05/16/2016).

## Background

Due to optimized therapies, life expectancy of patients with metastasizing cancer will continue to rise. Bone metastases affect 10–30% of all cancer patients, especially those located in the vertebral column. The main clinical problems are severe back pain and an increased risk of pathological fractures. Therefore, optimized palliative treatments to improve quality of life are necessary.

External beam radiotherapy (EBRT) is a well-established treatment option for patients with spinal metastases. EBRT results in adequate pain relief with acceptable local tumor control [1]. Of the retrospective series that described pain outcome mentioned in the systematic review by Gerszten et al., roughly 70% of patients had improvement in pain after radiation therapy [1]. The mean rate of local control was 77% (range, 61–89%). Although EBRT is the standard treatment for painful vertebral metastases, neither the optimal fractionation schedule nor the optimal standard dose have been established yet [2].

The two most common schemes are either 10 fractions of 3 Gy (30 Gy total dose) or a single-shot irradiation with 8 Gy [3]. Although EBRT is tolerated well and local control rates are acceptable, pain relief occurs delayed (within weeks) after EBRT, which in the light of the short life expectancy is unsatisfactory [4]. Based on data from the Bone Pain Trial [5], one can assume that as few as 20% of patients will experience pain relief within the first weeks.

Balloon kyphoplasty is a valuable treatment option for patients with painful and instable metastases to the vertebral column. In the CAFE trial [6], 134 patients with vertebral compression fractures were enrolled and randomly assigned to kyphoplasty or non-surgical management. The study found that patients treated with kyphoplasty had a significant reduction of back pain (from a numeric rating score [NRS] of 7.0 at baseline to 3.5 after kyphoplasty) after seven days.

However, kyphoplasty alone has no documented anticancer effect and thus EBRT is required to avoid early tumor regrowth. As this may take another 2–4 weeks (due to reduction of single doses to account for wound healing, e.g. 30Gy in 15 or 40 Gy in 20 fractions), this prolonged treatment poses an unacceptable mental and physical challenge to patients. These considerations also apply for 1times 8Gy, albeit not that distinct. Moreover, patients with progressive or simultaneous (e.g. visceral) metastases may also urgently require chemotherapy, which cannot be applied concurrently to radiotherapy due to the risk of potentiated toxicity.

To reduce treatment time and at the same time increase quality of life, novel techniques like kyphoplasty combined with (physical) anticancer methods are required. Our group developed a novel approach to deliver intraoperative radiotherapy during kyphoplasty (Kypho-IORT) [7–10]. In a recent dose escalation and cohort expansion phase I/II trial we enrolled 61 patients aged 50 years and older with a Karnofsky Performance Status of at least 60% and with one to three painful vertebral metastases confined to the vertebral body [11]. We detected that, after Kypho-IORT, the median pain score significantly dropped from 5 preoperatively to 2 at the first postoperative day (*p* < .001) and a persistent pain reduction beyond the first postoperative day of ≥3 points in 34 (79.1%) patients. As the 3, 6, and 12 month local progression-free-survival (L-PFS) was excellent with 97.5, 93.8, and 93.8%, we set up this randomized phase III trial to investigate whether this new technology may have the potential to replace the current standard of care.

## Methods/design

### Study design

**Fig. 1:**
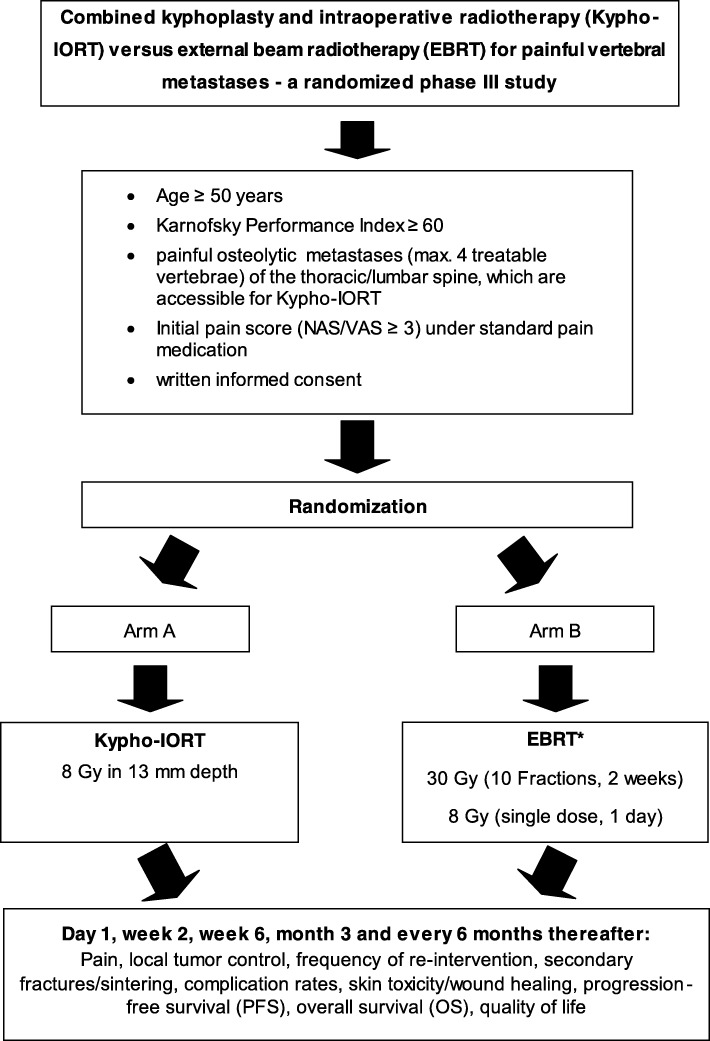
Trial Workflow. *study centers may choose between fractionated EBRT (30 Gy in 10 fractions) or single-dose EBRT (1 × 8 Gy). Once an option is chosen, the center must adhere to it for all patients included into the trial. Legend: IORT- intraoperative radiotherapy; Kypho-IORT – kyphoplasty or vertebroplasty + IORT; EBRT – external beam radiotherapy

This protocol (Version 2.0, April 2016) describes a prospective, multicenter, randomized phase III study set up to assess pain reduction after Kypho-IORT (Arm A) or EBRT (Arm B) for spinal metastases (Fig. 1). Patients will be recruited over a period of 2.5 years and followed to a maximum of 5 years.

### Objective and primary endpoint

The primary objective of this study is to test if the analgesic efficiency of Kypho-IORT is superior to standard-of-care EBRT in patients with painful vertebral metastases. The primary end point is to evaluate the percentages of patients with a pain reduction of at least 3 points (VAS) at the first post-interventional day (i.e. one day after Kypho-IORT in Arm A and one day after the begin of fractionated or single-shot EBRT in Arm B).

### Secondary endpoints

Secondary objectives are

- percentage of patients with a pain reduction of at least 3 points on VAS at week 2 and 6 weeks after start of treatment
- local tumor control, measured by longitudinal CT/MRI scans done at FUs by assigning one of the following lesion states: stable, osteoradionecrosis, pathological fracture, sintering progression, local relapse
- frequency of re-intervention or salvage therapies to the treated site
- secondary fractures/sintering progression
- overall progression-free survival
- adverse events
- incidence of cement leakage (including direction of leakage; symptomatic vs. asymptomatic)
- skin toxicity/wound healing, documented by regular imaging of the scar
- quality of life, assessed by completion of EORTC QLQ C30 and BM22 questionnaires

### Patient eligibility

Patients ≥50 years of age with up to 4 treatable vertebral metastases and a localized pain of VAS ≥ 3/10 are eligible to participate.

#### Inclusion criteria

- Age ≥ 50 years
- Karnofsky Performance Index ≥60
- initial pain score (VAS) of at least 3 under standard pain medication (change of the pain medication will me monitored)
- documented history of cancer (i.e. histological confirmation of the primary tumor)
- painful osteolytic metastases involving a maximum of 4 vertebrae of the thoracic/lumbar spine, which are accessible for Kypho-IORT
- written informed consent

#### Exclusion criteria

- any prior treatment (irradiation, surgery) of the target lesion(s)
- Lesions cranial to T3 (T1–2 and cervical spine)
- Tumors infiltrating dorsal vertebral structures (pedicles, lamina) or intraspinal (epidural) extension
- pathologic fracture with sintering of > 50%
- purely osteoblastic/osteosclerotic metastasis
- high likelihood for cement leakage (e.g. erosions of the bony borders of the vertebrae), which cannot be controlled otherwise by operating surgeon
- Pregnancy/lactation

### Screening and randomization

After informed consent, all patients will be screened for inclusion and exclusion criteria and obtain a patient ID. A baseline visit will then be performed, which includes the assessment of the KPS and VAS scores, comorbidities and medication, a gross neurological exam, the documentation of the underlying primary cancer and the completion of the QoL questionnaires to obtain baselines. If not performed before, a CT or MRI scan of the spine will have to be performed. After fulfilment of all inclusion criteria and in the absence of exclusion criteria, patients will then be allocated into any of the two arms by block randomization (block sizes of 2 or 4 and stratification by institute). Patients randomized into Arm A will undergo Kypho-IORT, patients randomized into Arm B will undergo standard EBRT (10 × 3 Gy or 1 × 8 Gy).

The intervention (Arm A or B) should begin within a time frame of 14 days after randomization.

### Arm a: Kypho-IORT

Kypho-IORT will be carried out under general anaesthesia as extensively described before [8–11]. All patients will be placed in prone position on a radiolucent table. An intraoperative low-energy x-ray device (Intrabeam®, Carl Zeiss Meditec AG, Oberkochen, Germany) will be used for intraoperative radiotherapy, the centre of the metastasis shall be chosen for the isocenter of the radiation.

The cement augmentation can be performed either as kyphoplasty or in a vertebroplasty technique. Each patient must receive early post-operative imaging of the treated vertebra to assess any leakage of cement. The direction of leakage and the clinical symptoms associated (symptomatic vs. asymptomatic) must be documented at this stage.

### Arm B: external beam radiotherapy

Patients randomized into arm B will receive conventional external beam radiotherapy, either as fractionated radiotherapy with 30 Gy delivered in 10 fractions of 3 Gy or as single-dose irradiation with 8 Gy. Each center must select and adhere to one treatment option (fractionated or singe-dose irradiation). Radiotherapy can be given as per established local standards (3D-conformal radiotherapy or intensity-modulated radiotherapy, local standards for target volume and risk organ delineation, local dose constraints). Patients will need to be seen at least once weekly by physicians and evaluated for adverse reactions.

### Assessment of efficacy parameters

#### Pain

Pain will be assessed at baseline (screening) and then at day 1 (first post-operative day in arm A, first day after treatment begin in arm B), week 2, and week 6 week after treatment and at each follow-up visit there will be an evaluation of pain intensity measured by the visual analogue scale (VAS) together with the current pain medication. Any re-intervention or salvage therapies to the treated site will be also documented.

#### Local tumor control, progression-free survival

At each follow-up visit (after 6 weeks, 3 months and thereafter each 6 months), there will be either a MRI or CT scan of the (complete) spine to determine the response of the treated lesion (stable, osteoradionecrosis, pathological fracture, sintering progression, local relapse) and to monitor for new metastases to other regions of the spine (we recommend the same diagnostic routine, which led to the treatment). Local control is defined as time span between randomization and local progression. Local progression is assessed either by serial CT or MRI scanning. Indicators for local progression in CT scans are:

- increase in size of osteolytic areas
- development of epidural disease
- progressive sintering/fracture of the treated vertebral body

Once progression is suspected in CT scans, a contrast-enhanced MRI should be performed, whereas indicators of progression are

- increase in the T2-signal
- epidural disease

It has to be considered that MRI and PET may show false-positive signals when the time to surgery is less than 6 months, whereas an epidural disease is the clearest indicator. When in doubt the PI and the steering committee have to decide on the case.

Overall progression-free survival is defined as time from randomization to any (confirmed) tumor progression or death by any cause.

#### Assessment of adverse events

At day 1, week 2, week 6, month 3 and each 6 m follow-up visit, a physical examination of the surgical scar /irradiation field will be performed to grade any of the following toxicities: dermatitis radiation, wound infection, scar, pigmentation, telangiectasia or other skin toxicity. In case of any skin toxicities, a photo will be acquired. A neurological examination will be performed to detect paresthesia, sensory numbness, motor weakness and paresis. An adverse event (AE) is then defined as any unfavorable and unintended sign (including an abnormal laboratory finding), symptom, or disease temporally associated with the use of a medical treatment or procedure regardless of whether it is considered related to the medical treatment or procedure (attribution of unrelated, unlikely, possible, probable, or definite will be interrogated for at each contact between the responsible investigator and the study subject until the end of the study). Ongoing AEs will be followed until recovery. Wherever possible, adverse events will be reported on the basis of CTC-AE v4.0.

A pre-existing disease or symptom (including pathological laboratory values) will not be considered an adverse event unless there will be an untoward change in its intensity, frequency or quality. This change will be documented by an investigator.

#### Serious adverse events (SAE)

Serious adverse events (SAEs) are defined as any event that is fatal, life threatening, causes or prolongs hospitalization; causes disability or incapacity or requires medical intervention to prevent permanent impairment or damage, or any grade 4 toxicity. Only SAEs that are judged to be related to surgery or irradiation by the local study center are reported.

In case of death an autopsy should be done to clarify the cause of death.

#### Quality of life

QoL will be determined using the EORTC quality of life C30 and the bone metastasis module questionnaire (EORTC-QLQ-C30/BM22). The questionnaires should be completed before.

treatment and during follow-up visits. The questionnaires have 30 respectively 22 questions and require approx. 10 min to complete. Even if the patient progresses or recurs before the last scheduled QoL assessment, QoL forms should still be completed.

### Statistical considerations

#### Trial sample size

This study is designed to test if the analgesic efficiency of Kypho-IORT is superior to standard-of-care EBRT in patients with painful vertebral metastases by comparing percentages of patients with a pain reduction of at least 3 points on VAS at defined points in time (day 1, week 2 and 6 after start of treatment).

Assuming a pain reduction of VAS-3 points at day one (first day after Kypho-IORT, one day after the first EBRT) for 40% of the patients after Kypho-IORT vs 5% of the patients after EBRT under standard pain medication (no change of the pretherapeutic pain medication 12 h after Kypho-IORT or 24 h after the first EBRT), the study will require 22 patients in each arm in order to have a two-sided alpha of 0.05 and a power of 0.817. Including a dropout rate of 20% at least 54 patients have to be recruited. The effect size was estimated on the basis of the results of the preceded phase I/II trial [11] and the results of RTOG 9714 [3].

#### Data analysis

Data will be analysed on an intention to treat basis. All variables will be described by descriptive statistics (frequencies, means and standard deviation, medians and minimum, maximum, depending on data distribution). The Chi-square test or Fisher’s exact test will be used for categorical data. For continuous data, parametric or non-parametric mean comparison tests will be used. The Kaplan-Meier method with log-rank tests will be used to estimate relapse-free survival and overall survival.

#### Ethics and informed consent

The trial will be carried out in compliance with the protocol, the principles laid down in the declaration of Helsinki, version as of October 1996 (as long as local laws do not require to follow other versions), in accordance with the ICH Harmonised Tripartite Guideline for Good Clinical Practice (GCP), the harmonized standards for Medical Devices (ISO 14155) and all other applicable regulatory requirements (local, regional and global). Standard medical care (prophylactic, diagnostic and therapeutic procedures) remains in the responsibility of the treating physician of the patient. This study was approved by the local ethics committee (Medical Ethics Commission II of the Faculty of Medicine Mannheim, University of Heidelberg, 2013-593 N-MA) and the Federal Office of Radiation Protection (Z5–22462/2–2013-116). All patients participating in the study need to provide written informed consent at least 24 h prior to the intervention.

### Trial registration

The trial is registered with ClinicalTrials.gov, number NCT02773966.

### Discontinuation of the trial

#### Individual withdrawal

Treatment of the patient must be discontinued if one or more of the following criteria apply:

- revocation of the patient’s declaration of informed consent
- lack of compliance
- discontinuation of the therapy for other reasons deemed necessary by the responsible physician (e.g. unfavorable intraoperative geometry)
- Grade IV skin toxicity, i.e. skin necrosis that require surgical intervention
- Grade IV bone toxicity, i.e. aseptic bone necrosis that require surgical intervention
- Grade IV nervous system toxicity, i.e. paresis

When therapy is discontinued, the reasons for discontinuing patient participation in the study must be listed in the patient’s file.

#### Investigator withdrawal

The steering committee is allowed to close the study ahead of schedule.

Other termination criteria include:

- insufficient patient recruitment
- unexpectedly severe toxicities (second Grade IV toxicity within the study)
- unexpected findings which prevent the continuation of the study for ethical and/or medical reasons (new benefit/risk analysis)

## Discussion

Kypho-IORT is a technically feasible new treatment option that provided immediate pain relief, structural stability and excellent local tumor control without severe side effects in a phase I/II trial [11]. As the potential benefit of this one-stop-procedure is still unclear in comparison to the gold standard (usually 30 Gy in 10 fractions in European countries, 8 Gy single shot in the US), we designed the randomized trial introduced here. The primary endpoint of this trial is pain reduction immediately at day 1 after treatment (one day after Kypho-IORT or one day after the first/only session of EBRT, respectively). We believe that immediate pain control is a key expectation for the vast majority of patients that are referred to radiation oncology for palliation of pain.

EBRT is an integral part of local therapy, but, as seen in several studies pain control is achieved in weeks (or even months) [12]. In the initial phase I/II trial, 70% of patients that had a preoperative pain score of ≥3 reported a reduction of 3 or more points at the first postoperative day [11]. The trial presented here will thus now evaluate whether this procedure may prospectively be offered as an (potentially superior) alternative to standard external-beam radiotherapy.

## Abbreviations

CRF: Case report form
CT: Computer tomography
CTC: Common toxicity score
EBRT: External beam radiotherapy
GCP: Good clinical practice
ISF: Investigator study file
Kypho-IORT: kyphoplasty/vertebroplasty + intraoperative radiotherapy
MRI: Magnetic resonance tomography
NRS: Numeric rating score
SAE: Serious adverse effects
VAS: Visual analogue scale
